# Immune checkpoint molecules in prevention and development of asthma

**DOI:** 10.3389/fimmu.2023.1070779

**Published:** 2023-02-14

**Authors:** Zahra Kanannejad, Saeede Soleimanian, Zahra Ghahramani, Najmeh Sepahi, Milad Mohkam, Soheila Alyasin, Nasim Kheshtchin

**Affiliations:** ^1^ Allergy Research Center, Shiraz University of Medical Sciences, Shiraz, Iran; ^2^ Hematology Research Center, Shiraz University of Medical Sciences, Shiraz, Iran; ^3^ Department of Immunology, School of Medicine, Shiraz University of Medical Sciences, Shiraz, Iran

**Keywords:** immune checkpoint, asthma, co-stimulatory signals, co-inhibitory signals, autoimmunity

## Abstract

Allergic asthma is a respiratory disease initiated by type-2 immune responses characterized by secretion of alarmins, interleukin-4 (IL-4), IL-5, and IL-13, eosinophilic inflammation, and airway hyperresponsiveness (AHR). Immune checkpoints (ICPs) are inhibitory or stimulatory molecules expressed on different immune cells, tumor cells, or other cell types that regulate immune system activation and maintain immune homeostasis. Compelling evidence indicates a key role for ICPs in both the progression and prevention of asthma. There is also evidence of asthma development or exacerbation in some cancer patients receiving ICP therapy. The aim of this review is to provide an updated overview of ICPs and their roles in asthma pathogenesis, and to assess their implications as therapeutic targets in asthma.

## 1 Introduction

Asthma is a respiratory disease initiated by crosstalk between the innate and adaptive immunity (1); however, T helper 2 (Th2) cells play a crucial role in driving airway inflammation and bronchial hyper-responsiveness, due to secretion of type-2 cytokines including interleukin-4 (IL-4), IL-5, and IL-13 (2, 3). Various effectors and signaling pathways contribute to the dissemination of allergic inflammatory responses that occur in epithelial, and stromal cells of the lung in allergic patients. Asthma is triggered by disruption of epithelial barrier integrity through environmental threats, such as airborne allergens. Consequently, epithelial cell (EC) injury results in the secretion of cytokines (TSLP, IL- 33, IL-25, and GM-CSF) and chemokines (TARC, CCL22, and CXCL10) from ECs. The release of alarmins promotes the stimulation of Th2 cells, type 2 innate lymphoid cells (ILC2s), and other immune cells such as macrophages, basophils and eosinophils (4). However, investigations reveal that other cytokines such as TGF-β1, which is involved in tissue remodeling during airway inflammation, also contributes to the pathogenesis of asthma through induction of Th2 and Th9 and suppression of Treg cells (5). In contrast, IL-37 down-regulates asthmatic airway inflammation by counterbalancing the disease-amplifying effects of IL-1β and IL-33 (6).

Asthma is a heterogeneous inflammatory disease characterized by complex pathophysiological pathways (7). Allergen- specific immunotherapy (AIT) through administration of increasing doses of relevant allergens to an allergic subject is the only disease- modifying treatment available for several common allergic conditions, such as asthma (8). Several biomarkers have been suggested by different studies as predictors of treatment efficacy, such as generation of allergen- specific IgG or IgG4 and IgA in serum (9, 10), suppression of local numbers of Th2/ILC2 cells or secretion of type 2 cytokines (11, 12) increased numbers of Th1 cells, Tregs, or Bregs (13–15), and induction of a recently introduced anti-inflammatory mediator, secretoglobin 1A1 (16), in the local environment. Various molecular effectors have been identified as therapeutic targets for severe asthma. For example, anti-asthma monoclonal antibodies against pro-allergic immunoglobulin E (IgE), IL-5, IL-4, and IL-13 have been developed. Since the regulation of T helper immune responses depends on the interaction between surface receptors and their ligands, inhibitory receptors could be considered as proper targets for asthma therapies (17, 18).

**Figure 1:**
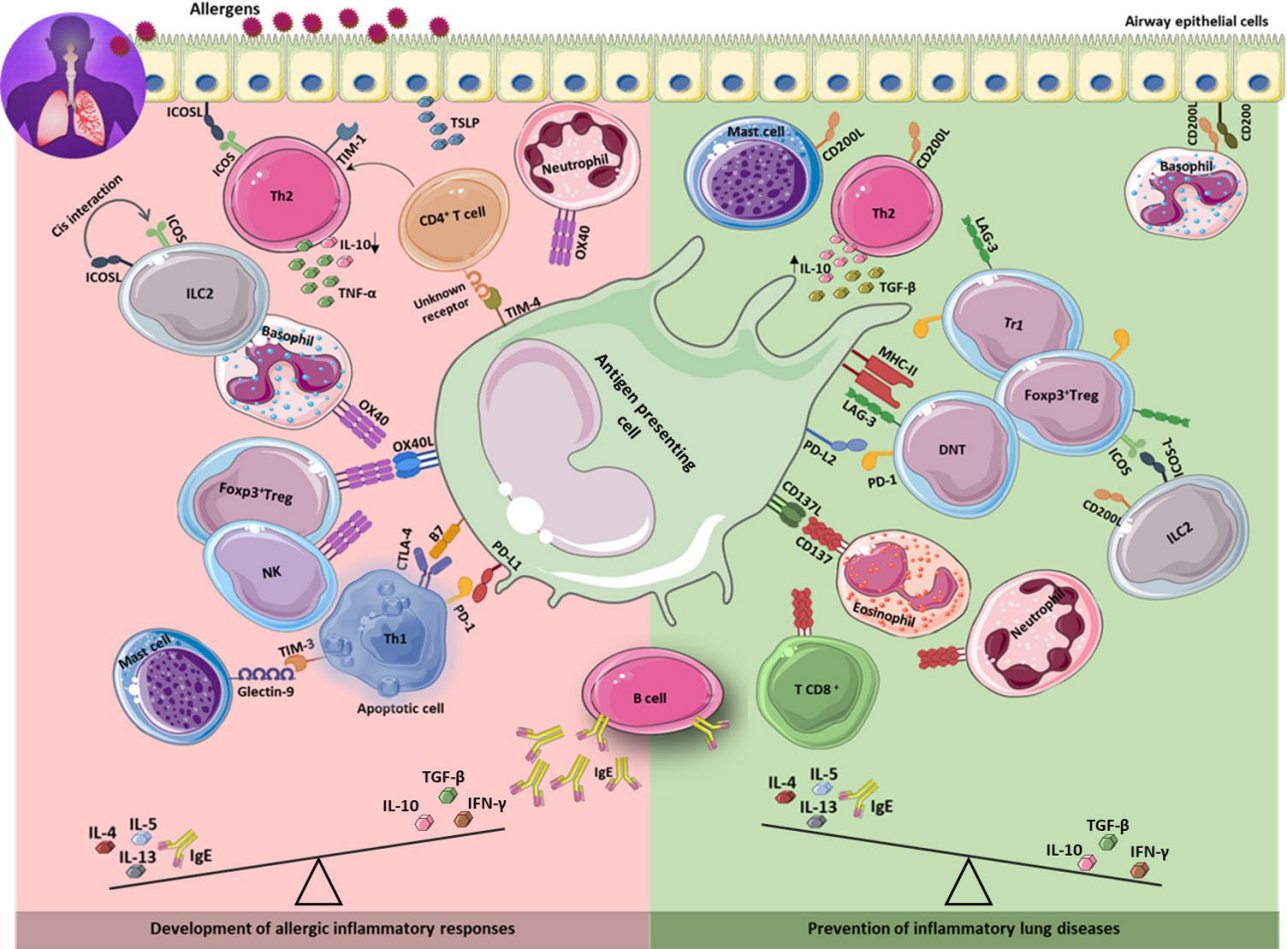
Immune checkpoints in the development and prevention of asthma. The development of asthma is associated with increased Th2 response results in secretion of IL-4, 5, 13, and IgE production. Inhibitory and stimulatory immune checkpoint molecules expressing on different immune cells as well as on lung epithelium, contribute to development (left) or prevention (right) of allergic inflammatory responses through interaction with their ligands on different cell types including APCs. Th2: T helper 2; IL: interleukin.

Immune checkpoints (ICPs) are inhibitory or stimulatory molecules expressed on the different immune cells, tumor cells, or other cell types that regulate immune system activation and maintain immune homeostasis. To date, many ICP molecules have been discovered including programmed cell death receptor-1 (PD-1), lymphocyte activation-gene-3 (LAG-3), T- cell membrane protein-3 (TIM-3), cytotoxic T- lymphocyte antigen-4 (CTLA-4), inducible co-stimulator (ICOS), and B7-H3. Stimulatory ICPs play important role in naïve T cell activation by providing the second signal (co-stimulatory signal), while signaling through inhibitory ICPs can direct activated T cells into a state called ‘exhaustion’ that is associated with reduced effector function and sustained expression of ICPs (19). Many pathogens and cancer cells can promote the inhibitory interactions to escape immune responses (20–26). Compelling evidence indicates a key role for ICPs in both progression and prevention of asthma (**Table 1**). Signals derived from costimulatory ICPs (for example CD28, ICOS, and OX40) together with IL-4, most likely derived from basophils, are required for initiating antigen- dependent polarization of naïve T cells toward Th2 cells and follicular helper T cells (T_FH_) (56). In contrast, antigen recognition in the presence of tolerogenic signals such as IL-10 and TGF-β leads to the development of telegenic dendritic cells (DCs) that promotes T regulatory cells (Tregs) development (57). Tolerogenic DCs may induce naïve T cell differentiation into Treg cells by providing inhibitory (PD-1 – PD-L1) and stimulatory (for example ICOS – ICOS-L) molecular interactions. Treg cells then inhibit sensitization and airway hyperresponsiveness through production of immunosuppressive cytokines (IL-10, TGF-β, and IL-35) and expression of inhibitory molecules (for example CTLA-4, PD-1, and LAG-3) and cytotoxic enzymes (granzyme B) (58). There is also evidence of asthma development or exacerbation in some cancer patients receiving ICP therapy. Based on the above considerations, the aim of this review is to provide an updated overview of ICPs and their roles in asthma pathogenesis (summarized in **Figure 1**), and to assess their implications as therapeutic targets in asthma.

**Table 1:** Effects of co-inhibitory/co-stimulatory immune checkpoints in asthma.

Checkpoint Molecule	Target cell	Possible effect on target cell in asthma
**ICOS**	Th2	Increased IL-4/IL-13 production (27–32)
	B cell	Increased IgE antibody class switching (31)
	ILC2	Induction of homeostatic survival and function of ILC2 (33, 34)
	Th17	Decreased IL-17 production and IL-17-dependent AHR (35)
**OX40**	Th2	Involved in development of pathogenic Th2 cells through TSLP (36–38)
	B cell	Correlated to total serum IgE (39)
**CD137**	Th2	Decreased Th2 proliferation and function (40)
	Th17	Regulation of the Th17/Treg balance (41)
	Treg	Regulation of the Th17/Treg balance (41)
	Mast cell	Enhanced cytokine production (42)
**CTLA4**	Th2	Increased IL-4/IL-13 production (43)
	B cell	Increased IgE antibody class switching (21)
**PD-1/** **PD-L1**	Th2	Increased production of related cytokines (44)
	Th1	Decreased production of related cytokines (44)
	Th17	Decreased production of related cytokines (44)
	Treg	Regulation of the Th17/Treg balance (45)
**CD200R**	Alveolar MΦ	Suppression (46)
	DC	Suppression (46)
	ILC2	Decreased activation and production of type 2 cytokines (47)
	Mast cell	Reduced degranulation (48)
**TIM-1**	Th2	Activation (49)
**TIM-3**	Th1	Suppression (50)
	Mast cell	Activation (51)
**Tim-4**	Th2	Development (52, 53)
**LAG-3**	Treg	Increased suppressive activity (54, 55)

ICOS, Inducible co-stimulator; CTLA4, Cytotoxic T lymphocyte antigen-4; PD1, Programmed cell death receptor-1; TIM, T cell/transmembrane, immunoglobulin, and mucin; LAG-3, lymphocyte activation-gene-3; MΦ, Macrophage.

## 2 Involvement of co-stimulatory receptors in asthma

### 2.1 Inducible co-stimulator (ICOS)

The inducible co-stimulator (ICOS), known as the third member of the CD28 family (59), is an activating co-stimulatory immune checkpoint expressed on activated T cells as well as Treg cells (60, 61). The ligand for ICOS, ICOSL (also known as B7-H2), is constitutively expressed on antigen- presenting cells (APCs), including B cells, DCs, and macrophages, and is downregulated upon binding to ICOS as an immunoregulatory mechanism (62). Its expression can be induced in non-lymphoid tissues such as lung epithelial cells and certain fibroblasts (63, 64). Interactions between ICOS and ICOSL trigger the activation of diverse signaling molecules that prevent apoptosis and play important roles in the development of memory and effector T cells and specific humoral immune responses. As discussed below, ICOS is critical for Th2 differentiation and function and for development of pulmonary inflammatory responses (27, 65).

Numerous studies using animal models with disrupted ICOS genes and antibody-blocking experiments have revealed the role of ICOS in tolerance induction. Preliminary studies in mouse models of Th1-mediated autoimmune diseases, including experimental autoimmune encephalomyelitis (EAE) and type 1 diabetes, using anti-ICOS mAbs have demonstrated disease exacerbation *via* disruption of the balance between effector and regulatory T cells (27, 66, 67). In allergy and asthma as Th2-mediated diseases, on the other hand, ICOS signaling regulates the magnitude of Th2-mediated pulmonary inflammation and clonal expansion (28–30). Functional studies have shown that ICOS-deficient mice exhibit profound defects in germinal center formation, antibody class switching and IL-4/IL-13 production (27, 31, 32). Similarly, in the context of allergic airway inflammation, ICOSL deletion leads to decreased serum IgE levels and inflammatory airway infiltration (68). Interestingly, a study using OVA-TCR transgenic mice showed that challenge with respiratory allergens leads to the induction of respiratory tolerance through development of Treg cells. This observation was at least partly, attributed to ICOS co-stimulation signals provided by pulmonary DCs suggesting a role for ICOS in respiratory tolerance (69).

Emerging evidence has revealed an interaction between ILC2 and Treg cells in allergic asthma, which involves ICOS: ICOSL ligation. The co-expression of ICOS and ICOSL on ILC2s has been demonstrated, and cis interaction of ICOS and ICOSL on ILC2s stimulates homeostatic survival and function of these cells (33, 34). Furthermore, induced Tregs (iTregs) have been shown to suppress ILC2-driven pro-inflammatory cytokines and pulmonary inflammation. This effect apparently involves Tran’s interaction of ICOS-L on ILC2 with ICOS on Tregs, which leads to decreased function of ILC2 *via* disruption of cis interaction of ICOS: ICOSL on ILCs. However, the authors also pointed out that iTreg-TGF-β signaling is the main mechanism of this suppression, and cell-cell contact through ICOS:ICOSL interaction is necessary for iTreg-mediated suppression (33, 34). Furthermore, suppression of IL-17 production by IL-35 producing ICOS^+^ Tregs and reversal of established IL-17-dependent AHR in mice is another example of ICOS involvement in controlling allergic asthma (35).

### 2.2 OX40

OX40 (CD134, TNFRSF4) belongs to the tumor necrosis factor receptor (TNFR) superfamily, which is predominantly expressed on activated CD4+ and CD8+ T cells (70–72). OX40 acts as a co-stimulatory receptor essential for regulating activation, survival, and expansion of conventional CD4+ and CD8+ T cells (73). It has been shown that the expression of OX40 is not restricted to activated conventional T cells but is also expressed on activated Treg cells, neutrophils and NK cells (74). The engagement of OX40 by its ligand OX40L (CD252) on antigen- presenting cells enhances T cell- mediated immune responses and contributes to the development of autoimmune diseases (75). Animal studies have revealed that OX40L is present in both asthmatic and non-asthmatic airway smooth muscle cells with higher levels of both OX40 and OX40L in asthma (76, 77). There is also an association between the expression level of OX40/OX40L and severity of asthma, since elevated expression was observed in bronchial submucosa of mild asthma, which was related to the accumulation of eosinophils and the level of IL-4 expression in the lamina propria (36). Another study investigated OX40L expression in atopic and non-atopic asthmatics, and found significantly higher levels of this molecule in atopic asthma. It was also shown that OX40L over expression in atopic patients was correlated with the specific hallmarks of the allergic asthma including total serum IgE suggesting a critical role for OX40L in the atopic mechanism (39). Indeed, involvement of OX40L in the induction phase of asthma has been suggested because the administration of a neutralizing antibody against OX40L prior to sensitization of mice prevented the induction of asthmatic responses with no effect on asthmatic responses during the challenge period. These observations provide evidence of the contribution of OX40 to the development of pathogenic Th2 cells, but not to the migration and activation of pathogenic Th2 cells in the lungs (78). OX40L has also been shown to be involved in development of Th2 responses to TSLP. TSLP activates human myeloid DCs to induce OX40L which triggers an inflammatory Th2 response characterized by high levels of TNF-α and low levels of IL-10 production, representing pathogenic Th2 cells involved in allergic inflammatory diseases such as asthma (36–38). Interestingly, exosomes derived from TSLP-activated DCs can induce differentiation of CD4+ T cells into Th2 cells in an OX40L-dependent manner (79). High expression of OX40L by activated basophils has also been demonstrated in a mouse model of allergic asthma. Blockade of OX40-OX40L interaction resulted in repressed differentiation of Th2 cells primed by basophils *in vitro* and attenuated OVA-induced eosinophilic inflammation in an experimental model (80). Such observations reveal essential modulatory functions of OX40–OX40L interactions in the development of allergic asthma, making them attractive candidates for clinical intervention.

### 2.3 CD137

CD137 (also known as 4-1BB) is a prototype co-stimulatory molecule for T cells (81). Treg cells are the only cell type with high levels of constitutive expression of CD137, while CD137 is expressed in lymphoid cells, including T cells of both CD4+ and CD8+ subsets, natural killer (NK) cells, NKT cells and myeloid lineage cells (82). Specific expression of CD137 has also been found in eosinophils derived from patients with allergic asthma or atopic dermatitis (83). Ligand for CD137 (CD137L) is a member of the TNF family and is expressed by APCs, which use it to co-stimulate T cell activity (84). The involvement of CD137/CD137L interactions in CD4+ T- cell-mediated inflammation has been demonstrated in various studies (81, 82). However, it is not clear whether this contribution involves activation of CD4+ T cells or other steps of inflammatory process (81). Moreover, these interactions have been suggested to be involved in the suppression of antigen-specific helper T cells and B cell-dependent humoral immune responses (83), and play an essential role in allergic asthma by regulating the Th17/Treg balance (41). It has been shown that administration of anti-CD137 in a mouse model of asthma not only prevented the development of asthma phenotype, but also completely reversed the established disease. Further investigations revealed the association of protection with reduced Th2 proliferation and function, as well as elevated secretion of the Th1-type cytokine IFN-γ (40). Confirming these observations, agonistic Abs to CD137 could directly co-inhibit Th2 responses and co-stimulate Th1 responses *in vitro*. However, CD137-mediated suppression of Th2 response is independent of IFN-γ (85). Cytokine production by mast cells following FcϵRI engagement was also enhanced in response to agonistic anti-CD137 antibodies, as demonstrated by another study (42). Interestingly, Stimulation of the CD137L using an agonistic antibody against CD137L instead of CD137 in an animal model of asthma prevented the development of asthma phenotype but failed to reverse the established disease. These results were partly attributed to IFN-γ-producing CD8+ T cells (86).

While multiple studies have indicated reduced airway hyper-reactivity, eosinophilic airway inflammation, mucus production, and IgE levels following administration of anti-CD137 mAb, indicating its potential to alleviate asthma (40, 87–90), a study using anti-CD137 antibody showed no reduction in airway hyper-reactivity and eosinophilic inflammation in a murine model of asthma, although abolished allergen-specific IgE was observed (91). Mice deficient in CD137 have also been shown to develop eosinophilic airway inflammation associated with Th2 cytokine production and allergen-specific serum IgE levels (92). In accordance with these observations, both soluble and membrane-bound CD137L levels in monocytes are reduced in patients with allergic asthma (41). Regarding the contribution of CD137 to the development of asthma, more experimental studies are required on the application of CD137 directed immunotherapies in the context of allergic diseases such as asthma.

## 3 Involvement of co-inhibitory receptors

### 3.1 Cytotoxic T lymphocyte antigen-4 (CTLA-4)

CTLA-4, also known as CD152, is a member of the B7/CD28 family and has a high affinity for B7-1/2. CTLA-4 is constitutively expressed on Treg cells, but is inducible in conventional T cells (93). CTLA-4 competes with CD28 for B7 binding and produces inhibitory signals that counteract stimulatory signals originating from CD28:B7 ligation (94). CTLA-4 blockade leads to an augmented immune response owning to the potentiation of helper T cell function. Conversely, engagement of CTLA-4 in Tregs enhances their suppressive activity (95). Disruption of this delicate balance in immune regulation may lead to autoimmune or atopic diseases (96). Multiple polymorphism studies have investigated the association between CTLA-4 and asthma severity and AHR, providing evidence for involvement of these polymorphisms in controlling helper T cell- mediated responses and total serum IgE levels in patients with asthma (97, 98). The surface expression of T cell co-stimulatory molecules CTLA-4 and CD28 and their counter-ligands is differentially induced for T cell activation and expansion in atopic asthma (99). Recent studies have indicated an elevation in plasma soluble CTLA-4 and CS28 and their counter ligands B7-1 and B7-2 in asthmatic patients (100, 101). Generally, sCTLA-4 levels are higher in atopic and non-atopic asthma as severity increases (99, 100, 102–104) and its concentration is positively and significantly associated with total serum IgE and eosinophil count in atopic asthmatic patients (21, 43, 104, 105). The exact role of these soluble co-stimulatory molecules in the pathogenesis of allergic asthma is not clear; however, it may be a consequence of the dysregulation of T cell activation (101, 104).

In a study of children with recurrent wheezing with high- or low-risk factors for asthma, a reduced absolute number and percentage of CD4+CD25+CTLA-4+ T cells was shown compared to healthy children. Additionally, stimulation of PBMCs with house dust mite extract resulted in decreased expression of CTLA-4 mRNA in high-risk children, suggesting a potential association between defective expression of CTLA-4 and development of asthma (105). Cumulative evidence suggests a potential therapeutic capacity of CTLA-4 in diverse autoimmune diseases and atopy. In accordance with this evidence, a high dose allergen-CTLA-4-encoding DNA vaccine was shown to suppress Th2 immune responses induced by allergens in a mouse model of asthma (43).

### 3.2 Programmed cell death protein-1 (PD-1)

Programmed cell death 1 protein (PD-1) is an inhibitory immune checkpoint molecule expressed by exhausted CD8+ T cells, activated T cells, naive and activated B cells (21). Signaling pathways triggered by the binding of PD-1 to its ligands, PD-L1 (B7-H1) and PD-L2 (B7-DC), are essential for regulating the balance between T-cell activation and tolerance, thereby contributing to the pathogenesis of various diseases such as autoimmunity, tumors, infectious diseases, and allergies (17, 106, 107). Primary role of PD-1 is the regulation of T cell responses *via* promotion of apoptosis in effector T cells and inhibition of apoptosis in Treg cells after antigen clearance (21). However, chronic engagement of TCR in the absence of costimulatory signals can cause PD-1 overexpression leading to anergy, exhaustion and tolerance of CD4+ and CD8+ T cells (108, 109). It has been revealed that ligation of PD-1 enhances production of Th2 cytokines by naïve T cell and decreases production of Th1 and Th17-related cytokines (44). Elevated expression of PD-1 and CTLA-4 in activated Th2 cells upon allergen exposure was observed in a mouse model of asthma. The long-term persistence of these exhausted pro-allergic Th2 cells during AIT with a reduction during up-dosing stage might explain the long-term treatment period in AIT to obtain clinical and immunological benefits (110). Involvement of PD-1 in pathogenesis of allergies has been investigated in several studies. It has been shown that altered PD-1/PD-L1 signaling might contribute to the balance between Th17 and Treg cells leading to development of asthma (45). In a mouse model of asthma sustained upregulation of PD-1 and PD-L2 has been observed in the lungs of asthmatic mice. Moreover, *in vivo* blockade of PD-L2 results in exacerbation of the disease due to elevated serum IgE levels, increased eosinophilic and lymphocytic inflammation, and elevated Th2 related cytokine production (111). These observations are in sharp contrast to the *in vitro* results with diminished proliferation of stimulated T cells and decreased production of Th2-related cytokines (111). Enhancement of AHR by PD-L2 blockade has also been illustrated by other investigations indicating a protective role of PD-L2 against the initiation and progression of airway inflammation (112, 113). In line with these observations, the results obtained from knock out animals showed opposing roles of PD-L1 and PD-L2 in asthma. While increased AHR, lung inflammation and Il-4 production by iNKT cells were observed in PD-L2^-/-^mice, PD-L1^-/-^ mice showed decreased AHR and lung inflammation and increased secretion of IFN-Y by NK cells (114). Taken together, an opposing role for PD-L1 and PD-L2 is suggested since PD-L2 deficiency results in a more severe disease, while mice deficient in PD-L1 experience reduced AHR and lung inflammation.

### 3.3 CD200R

CD200 receptor (CD200R) is an endogenous inhibitory signaling molecules that is mainly expressed in the lungs, predominantly by alveolar macrophages, neutrophils and mast cells (115). There are several isoforms of CD200R, among which CD200R1 is the best- characterized (116). CD200, a type-1 cell membrane glycoprotein, is the only known ligand of the CD200R family and is primarily expressed in pulmonary epithelial cells (115, 117). The CD200:CD200R1 inhibitory signaling pathway has been suggested as a mechanism to prevent an inflammatory response to harmless inhaled antigens. The activation of alveolar macrophages and DCs is tightly regulated by epithelial cells through the release of soluble mediators such as TGF-β, nitric oxide (NO), and IL-10, as well as their interactions with CD200 (118). Binding CD200 on airway epithelium to CD200R on alveolar macrophages and DCs initiates an inhibitory signal and thus represses activation of these cells preventing inflammatory lung diseases (46). It has been shown that the expression of CD200R1 is markedly up-regulated on alveolar macrophages, DCs and mast cells upon allergen exposure and/or in pulmonary infections (46, 119). In the context of influenza infection, previous reports have shown that alveolar macrophages in CD200-deficient mice are more active, leading to delayed resolution of inflammation and increased mortality (46). CD200R1 is also been found to be expressed in asthmatic inflammatory cells including basophils, Th2 cells, and ILC2 (115, 120). CD200R upon ligation prevents activation, proliferation and production of type 2 cytokines in activated ILC2s (47). Importantly, CD200R engagement on ILC2 using CD200-Fc chimeric protein in humanized mouse models of asthma dampened airway resistance, reduced eosinophilia and improved pulmonary dynamic compliance (47). Such observations provide evidence confirming regulatory role of CD200:CD200R as an important immunological checkpoint crucial for the maintenance of immune tolerance in the airway mucosa. The effect of CD200R engagement stimulation on AHR inhibition has also been attributed to local alterations in T cell responses and cytokine secretion (121). Other investigations have revealed that CD200:CD200R interaction diminishes allergic inflammation through reduced degranulation of mast cells and basophils (48, 122). Dysregulation of CD200 in asthma has been confirmed by several studies evaluating CD200 expression in PBMCs of asthmatic patients or serum levels of soluble CD200 which is probably produced by membrane shedding (123) or mRNA splicing (124) during asthma pathogenesis. However, the exact function of sCD200 in human diseases and its anti-inflammatory proprieties remain unclear. Further experimental investigations are needed to understand the cell specific functions of different isoforms of CD200R during homeostatic and inflammatory processes.

### 3.4 T cell immunoglobulin and mucin-domain containing protein (TIM) family

There has been a growing interest in the function of the T cell/transmembrane, immunoglobulin, and mucin (TIM) (gene family, which are the cell surface receptors for phosphatidylserine (PtdSer). These proteins with broad immune functions play essential roles in the regulation of innate and adaptive immune responses (125). Three members of TIM gene family (TIM-1, -3 and -4) are located on human chromosome 5q33 and have frequently been associated with asthma, allergy, and autoimmunity (126, 127). The molecules of TIM family are expressed on different lymphoid and myeloid immune cells, including T cells, B cells, NK cells, mast cells and macrophages (128, 129), and due to widespread manner of their function, the definition of TIM has been proposed to be *transmembrane* immunoglobulin and mucin” instead of “T cell/transmembrane”, immunoglobulin, and mucin (128). PtdSer on the surface of apoptotic cells is recognized specifically by immunoglobulin variable region domains of TIM-1, TIM-3, and TIM-4 which are expressed in mouse and human cells with different expression profiles, thereby playing distinct roles (126). TIM-1 is expressed on the surface of Th2 cells, generating a costimulatory signal that drives T cells activation in the context of asthma and allergy (49). TIM-3 is a co-inhibitory molecule that is preferentially expressed on Th1 and T-cytotoxic1 (Tc1) cells, and upon ligation to its ligand, promotes apoptosis of these cell populations (50). Therefore, there may be a reciprocal relationship between TIM 1 and TIM-3 in the regulation of Th1 and Th2 activity (126). TIM-4 is selectively expressed on APCs. Recent evidence indicates that the binding of TIM-4 to T cells *via* unknown receptors can promote the development of Th2 cells (52, 53). The immune response to infectious agents, tumor-specific antigens, and allergens results from a balance between Th1 and Th2 cells. The cross-regulation and differentiation of these subsets lead to two distinct scenarios; first, prevention of autoimmunity that results from the stimulation of Th2 cell cytokines and the second, regulation of asthma and allergic diseases that result from the stimulation of Th1 cytokines (130). Similarly, in the context of asthma and allergic rhinitis, the interaction between Tim-1 and Tim-4 causes the expansion of Th2 cells, mitigating asthmatic inflammation. In this scenario, Tim-3 is involved in the induction of Th2 cytokines (IL-4, IL-5, IL-13) through stimulation of mast cells (51). Consistent with this activity, TIM-3 by binding to its ligand, Galectin-9, stimulates apoptosis of Th1 cells thereby reducing the production of IFN-γ (128). Indeed, TIM-3, as a negative regulatory molecule, play a significant role in regulation of the balance between Th1 and Th2 which could affect the development of allergy and asthma. Thus, TIM3 upregulation in CD4+ T cells leads to a Th1/Th2 imbalance in allergic asthma patients. An *in vitro* study it was indicated that Tim-3 blocking dramatically reduced Th2-produced cytokines and promoted Th1-produced cytokines including TNF-α and IFN-γ in the supernatant of PBMCs. It has been shown that after co-culture of CD4^+^T cells and B cells, the production of IgG and IgE was decreased by blocking Tim-3 (51, 130). Therefore, Tim-3 blocking may be of therapeutic benefit in reducing airway inflammation by up regulating Th1 cytokines, dampening Th2 cytokines, and reducing IgG/IgE generation.

### 3.5 Lymphocyte activation gene-3 (LAG-3)

Lymphocyte activation gene-3 (LAG-3) is an inhibitory immune checkpoint expressed on various cell types, especially CD4^+^T cells, CD8^+^ T cells, NK cells, NKT cells, B cells, and Treg cells. Major histocompatibility complex-II (MHC-II) is a major ligand for LAG-3, and other ligands, including fibrinogen-like protein-1 (FGL-1), α-synuclein fibrils (a-syn), galectin-3 (Gal-3), and lymph node sinusoidal endothelial cell C-type lectin (LSECtin), have also been discovered. LAG-3 expression is induced upon TCR activation or by cytokines, especially IL-2, IL-12, IL-27, and IL-7. Once LAG-3 binds to its ligand, it inhibits T cell activation through its cytoplasmic inhibitory domains.

It has been shown that T regulatory type 1 (Tr1) cells express high level of LAG-3 for cell contact-mediated immune suppression during asthma (131). Activin-A has been reported to be the main cytokine for Tr1 generation and LAG-3 expression in allergic asthma (132). The adoptive transfer of LAG3+CD49b+CD4+ Tr1 cells improves atopic asthma (133). LAG3 is also essential for Foxp3+ Treg function, since LAG-3 deficient Tregs show reduced suppressive activity (54, 55). Li X et al., showed that IL-10 differentiated dendritic cells (DC10) of asthmatic individuals induced differentiation of CD25+Foxp3+LAG3+CTLA-4+ Tregs, which suppressed T effector response in a contact dependent manner (134). In addition to Tr1 and Foxp3+ Treg cells, ex vivo-generated double- negative T cells (DNT) exert an antigen-specific regulatory function through LAG-3 expression. Indeed, elevated expression of LAG-3 in DNT cells in a murine model of allergic asthma affected MHC-II antigen recognition and thus modulated allergen-specific immune regulation by these cells. Allergen-induced AHR, pulmonary inflammation, and allergen-specific IGE/IgG were inhibited (135). LAG-3 expression has also been reported in Treg-of-B cells, a subset of Treg cells which are induced from naïve T cells by follicular B cells in Peyer’s patches (136). LAG3 also has a crucial effect on the suppressive capacity of these cells. In a mouse model of asthma, the adoptive transfer of LAG3+ Treg-of B cells inhibited Th2 cytokine secretion and eosinophilic inflammation and ameliorated asthma symptoms (137). Elevated LAG-3 expression was also reported in lung B cells from sensitized mice in a murine model of asthma in a recent study by Habener et al. (138). This finding is in line with the reported expression of LAG-3 in immunosuppressive natural plasma cells in an animal model of infection (139), and the ability of LAG-3+ Tregs to suppress DC maturation and MHC-II-directed functions (140) indicates a crucial role for LAG-3 in the development and pathogenesis of asthma.

## 4 Immune checkpoint molecules as biomarkers in asthma

ICP molecules can be considered as predictive biomarkers for asthma susceptibility or severity. Numerous studies have reported a genetic association between susceptibility to asthma and ICPs. A meta-analysis study detected +49A/G polymorphism in CTLA-4 as an important risk factor for asthma susceptibility, especially in Asian individuals, children, and atopic patients (98). TIM-3 574T>G and TIM-1-416G>C single nucleotide polymorphisms are associated with an increased risk of asthma (128, 141). In addition to genetic associations, increased plasma concentration of CTLA-4 protein and cell expression of TIM-3 have been proposed as biomarkers for asthma susceptibility and severity in different studies (99, 102, 104, 142). Cellular expression level of some ICP can also be considered as biomarker of asthma. CD137 is increased in circulating eosinophils in IgE-mediated allergic asthma and is associated with increased IgE levels (143). In asthma, CD200 expression is reduced in peripheral blood cells of patients with asthma during exacerbation, and pharmacological modulation of this receptor has been shown to improve clinical and inflammatory outcomes in preclinical asthma models (144, 145).

Immune checkpoint inhibitors (ICIs) have been used for treatment of many different types of malignancies either as monotherapies or in combination with other treatments including chemotherapy and adjuvants showing remarkable clinical benefits (146). However, they are also linked to various immune-related adverse events (irAEs) caused by abnormal activation of autoreactive T cells with a prevalence of approximately 20–30% which can affect any organ in the body (147). Pulmonary toxicity is well documented with a wide range of toxicities reported and an overall incidence of 2.7–3.5% depending on the tumor type and treatment (148). Interestingly, lung toxicities appear to be more frequent with combination regimens than with monotherapies (149). The development of bronchial asthma or its exacerbations has also been reported in a few cases receiving ICI therapy (**Table 2**). However, there is a lack of knowledge regarding these types of irAEs, as only a limited number of cases have been reported. There are no established predictive markers for ICI-related toxicities. Peripheral blood eosinophilia, elevation of serum levels of total IgE, reversible airflow obstruction and elevated fractions of exhaled nitric oxide have been reported in these patients (150). Elevated eosinophil counts have been associated with an increased risk of toxicity and better clinical outcomes of ICI treatment in several types of advanced cancer (158–161). However, whether the proposed biomarkers have a specific predictive impact on ICI therapy outcomes is uncertain but, provides a foundation for future investigations in randomized controlled trials.

**Table 2:** Asthma as an IrAE in ICI-treated cancer patients reported in the literature.

Type of Cancer	Treatment	Subjects	irAEs	Marker	References
Adenocarcinoma	Nivolumab (3 mg/kg every 2 weeks)	50-year-old man	Asthma	Eosinophilia (11%), ↑ lgE (863 IU/mL)	(150)
Melanoma (Stage IV)	Ipilimumab and Nivolumab	34-year-old female	Asthma	Eosinophilia (up to 2.2 g/L) ↑ lgE (763 IU/L).	(151)
Squamous cell carcinoma of the lung (Stage IVA)	Durvalumab (1500 mg/body, every 3 weeks)	70-year-old man	Asthma	Eosinophilia (580 to 710), FEV1(1.59 L), FEV1/FVC (64.37%), FeNO (78 ppb)	(152)
Squamous cell carcinoma of the lung (stage IIIA)	Durvalumab	65-year-old man	Asthma	Eosinophilia (300cell/u), FEV1(1.44L), FEV1/FVC (50.17%), FeNO (47 ppb)	(152)
Kidney neuroendocrine carcinoma	Nivolumab (biweekly, total 200 mg/body)	65-year-old man	Asthma and sinusitis	Eosinophilia, abnormal bilateral shadow in chest CT, severe alveolar hemorrhage	(153)
Bladder cancer	Pembrolizumab	70-year-old female	Asthma	1 TIM-3 in CD4+ memory T cells, 1Th17, 1gE (291 IU/mL)	(154)
Malignant melanoma	IBI310 and Sintilimab	57-year-old woman	Asthma	Eosinophilia (17.30%)	(155)
Malignant melanoma (Stage IIIb)	Pembrolizumab	72-year-old male	Asthma	Eosinophilia (1.1x10^9^), FEV1(2.57L), FVC (4.04L), FEVI/FVC (64%), FeNO (129 ppb)	(156)
Lung adenocarcinoma (Stage IIIA)	Durvalumab (10 mg/kg every 2 weeks)	69-year-old male	Asthma	Eosinophilia (500cell/u)	(157)
Lung adenocarcinoma (Stage IIIB)	Durvalumab (10 mg/kg every 2 weeks)	71-year-old male	Asthma	Eosinophilia, FVC (5080 ml), FEV1(2270ml), FEVI/FVC (44.7%), FeNO (39.1 ppb)	(157)

IrAE, immune-related adverse events; FEV1, forced expiratory volume in 1 s; FVC, forced vital capacity; FeNO, fractional exhaled nitric oxide; TIM-3, T cell immunoglobulin and mucin-domain containing-3.

Owing to their success in cancer therapy, ICIs have gained considerable interest as treatments for other immune-related diseases (162). However, it is controversial ICI therapy would be beneficial in treatment of such diseases due to a lack of knowledge about exact compensatory mechanisms in different immune checkpoint signaling pathways. In addition, it is unclear whether the reported alterations in the expression of immune checkpoint molecules in asthma are pathogenic or an outcome of the disease. Given the complex nature of immune responses, particularly in the context of immune-related diseases such as asthma, further investigations on agonistic or antagonistic targeting of one or more immune checkpoint axes are needed to evaluate the outcomes with a more comprehensive approach.

## 5 The role of microbiota and probiotics on immune checkpoints

The microbiota can play various vital functions in the development of human immunity and the setup of immunological homeostasis (163). Lifestyle parameters comprising of hygiene, diet, encountering to bacteria or viruses, and medical intercessions with any medication, amend variability of phylogenetic composition and the cross- talk quality among adaptive and innate immune systems through skin and mucosal epithelia. Recently, several reports support the association of microbiota and their composition for offering protective impacts for healthiness (164). However, dysbiosis or alteration in microbial diversity has been associated with immune-related diseases including cancer and allergy, conditions that are accompanied by exaggerated or impaired immune tolerance, respectively (165). In this context, probiotics’ administration has been considered to modify this dysbiotic digression by reinstating the gap between the current and previous microbial communities.

Many functional mechanisms of probiotics or their derivatives (postbiotics) have been formerly described (166, 167). However, the exact mechanisms considering the function of the human microbiota remain unclear. The postulated mechanisms include reduction of mast cell infiltration, modulation of Th1/Th2 balance, and elevation of Treg population in peripheral lymph nodes (168, 169). In this section, we will discuss the possible interaction of microbiota/probiotics and immune checkpoints based on of new findings.

The formation of lung microbiota is crucial for the maturation of the immune system and for orchestrating respiratory function in health or disease (170). In this context, during the two first weeks of a neonatal mouse’s life, the drift of lung microbiota from Firmicutes and Gammaproteobacteria to *Bacteroides* was linked to the appearance of CD4^+^Helios^−^Foxp3^+^ Treg cells, which need the interaction with PD-L1 for progression in the lungs. Therefore, any dysbiosis or lack of microbiota as well as PD-L1 blocking leads to allergen responsiveness alongside adulthood (171). In another study, it was demonstrated that DCs of germ- free (GF) mice showed elevated levels of CD137, ICOSL, and OX40L than of controls in DCs. These differences in the activation status of the cells may direct the lung environment toward a Th2-biased immune response. Although, the precise function of DC populations or the associations of their activation status, in the course of allergic asthma stays unknown, it is obvious that the phenotype and number of DCs are affected by the commensal microbiota (172).

Overall, the knowledge of microbiota has provided new insights into pathogenesis of allergic diseases including asthma owing to the various immunomodulatory features of the mutualistic microorganisms. The microbiota effects can be indirect or direct *via* a cellular cross- talk with and between adaptive or innate immune cells, through the biomolecules exchanging which in turn could provoke inflammatory or regulatory cells. The consequent outcomes can be reconstitution or inflammation, with differing effects in allergy. However, the exact mechanisms of the possible role of microbiota in controlling allergic diseases, especially in the field of immune checkpoints are still unclear. Thus, additional investigations with a higher preference for identification of immune checkpoint functions in allergic diseases are of importance.

## 6 Conclusion

The balance between pro-/anti-inflammatory and the lung-resident immune cell network is of crucial importance in the maintenance of pulmonary homeostasis. The immune checkpoint molecules provide stimulatory/inhibitory signals during different stages of cellular and humoral immune responses. Ligation of CD28 by B7-1/-2 leads to a cascade of intracellular signaling that provides the second signal essential for naïve T cell activation and clonal expansion. CD28 costimulation is also required for B cell migration into the B cell follicles and provides proper signals for antibody production (173). Signals through an inhibitory immune checkpoint, ICOS, result in elevated IL-4/-13 secretion and thus increased IgE production by B cells in airways. In addition, CTLA-4 ligation by B7 molecules on DCs or activated lung epithelial cells, along with PD-1/PD-L1 interaction is involved in increased production of type-2 cytokines, IgE secretion, and formation of AHR. Other immune checkpoint molecules such as CD200 together with TNF receptor family members, CD137 and OX40, are also involved in the severity of pulmonary inflammation and AHR through signals that affect the balance of immune cells in the lungs. Indeed, the complex interactions between the signals through different immune checkpoints is necessary to limit the activation of type-2 immune responses and eosinophilic inflammation in pulmonary airways. Understanding these complex and comprehensive interactions not only provides insights into the pathogenesis of allergic asthma but also paves the way for setting up new strategies for therapeutic intervention in the context of allergic airway inflammation.

## Author contributions

All authors took part in literature search and manuscript preparation and editing. All authors contributed to the article and approved the submitted version.
